# Anemia and Its Associated Factors Among Children Aged 6–23 Months in Nine Sub‐Saharan African Countries: A Multilevel Proportional Odds Model Using the Latest Demographic and Health Survey (DHS) Data

**DOI:** 10.1002/hsr2.72693

**Published:** 2026-06-19

**Authors:** Temesgen Gebeyehu Wondmeneh

**Affiliations:** ^1^ Department of Public Health, College of Medical and Health Science Samara University Semera Ethiopia

**Keywords:** anemia, associated factors, children aged 6–23 months, demographic and health survey (DHS), proportional odds model, Sub‐Saharan Africa

## Abstract

**Background:**

Anemia remains highly prevalent among children aged 6–23 months in sub‐Saharan Africa. Previous estimates relied on outdated World Health Organization (WHO) hemoglobin cutoffs, which may have overestimated the burden. This study assessed anemia prevalence and associated factors using the updated 2024 WHO thresholds in nine sub‐Saharan African countries.

**Methods:**

A cross‐sectional analysis was conducted using recent Demographic and Health Survey data (2021–2024). A weighted sample of 14,480 children aged 6–23 months was included. A multilevel proportional odds model was applied to identify factors associated with anemia severity. Adjusted odds ratios (AORs) with 95% confidence intervals (CIs) were reported, and statistical significance was declared at *p* < 0.05.

**Results:**

The overall prevalence of anemia was 62.04% (95% CI: 61.3–62.8), including 2.94% severe, 31.96% moderate, and 28.14% mild anemia. Female children had higher odds of being in less severe anemia categories compared with males (AOR = 1.40, 95% CI: 1.27–1.45). Higher maternal education (secondary or higher: AOR = 1.37, 95% CI: 1.23–1.52), health insurance coverage (AOR = 1.74, 95% CI: 1.55–1.96), adequate antenatal care (AOR = 1.09, 95% CI: 1.01–1.17), deworming (AOR = 1.14, 95% CI: 1.06–1.23), and adequate dietary diversity (AOR = 1.25, 95% CI: 1.10–1.41) were associated with lower anemia severity. In contrast, fever (AOR = 0.75, 95% CI: 0.67–0.81), underweight status (AOR = 0.87, 95% CI: 0.78–0.97), and multiple births (AOR = 0.65, 95% CI: 0.49–0.85) were associated with more severe anemia.

**Conclusion:**

Anemia remains a major public health problem in the included countries despite the use of updated WHO thresholds. Interventions targeting maternal education, child nutrition, infection prevention, and access to healthcare services are essential to reduce anemia severity.

**Abbreviations**
AIC: Akaike Information Criterion
AOR: adjusted odds ratio
BIC: Bayesian Information Criterion
CI: confidence interval
DHS: demographic and health survey
FIG: Figure
HAZ: height‐for‐age z‐score
Hb: hemoglobin
ICC: intraclass correlation coefficient
MOR: Median odds ratio
PCV: Proportional change in variance
WAZ: Weight‐for‐age z‐score
WHO: World Health Organization
WHZ: Weight‐for‐height z‐score

## 1 Introduction

Anemia remains one of the most pervasive global public health problems worldwide, disproportionately affecting infants and young children in low‐ and middle‐income countries (LMICs) [1, 2, 3]. Anemia in early childhood has serious and lasting consequences. Iron deficiency anemia impairs oxygen delivery and is linked to deficits in cognitive and psychomotor development, growth, immunity, and increased morbidity and mortality [4, 5]. Anemia in the first 2 years of life may cause irreversible impairments in learning and later school performance, perpetuating cycles of poor health and poverty [6]. Children aged 6–23 months are particularly vulnerable because of rapid growth, increased iron requirements, and the transition from exclusive breastfeeding (EBF) to complementary feeding, which is often inadequate in quality and quantity [7, 8]. Globally, it is estimated that approximately 40%–45% of children under 5 years of age are anemic, representing nearly half a billion children [9, 10]. According to the Global Burden of Disease (GBD) 2021 estimates, anemia affected nearly 2 billion people worldwide, with preschool‐aged children bearing one of the highest burdens [11, 12]. Sub‐Saharan Africa bears the highest global burden of childhood anemia; Demographic and Health Survey (DHS) analyzes using traditional WHO hemoglobin cutoffs estimate a prevalence of 60%–64.1% among children aged 6–59 months [13, 14, 15] and 76.6% among those aged 6–23 months [16]. Marked variation exists across countries and sub‐regions, reflecting differences in socio‐economic conditions [17], dietary practices, disease burden, and access to health services [14, 18, 19]. Country‐specific studies from Ethiopia [20], Nigeria [21], and Ghana [17] report anemia prevalence ranging from 49% to over 75% in this age group. Despite numerous nutrition and child health interventions, progress in reducing childhood anemia in the region has been slow, underscoring the need for updated evidence to inform policy and programming.

Anemia in early childhood is a multifactorial condition influenced by complex interplay of nutritional, infectious, maternal, household, and community factors [13, 22]. Poor dietary diversity, food insecurity, and suboptimal infant and young child feeding practices substantially increase the risk of anemia among children under five [23]. Maternal characteristics such as anemia, low educational attainment, unemployment, inadequate antenatal care (ANC), and poor nutritional status further contribute to childhood anemia, particularly in rural areas [15, 20, 22, 24]. Higher maternal and community education, rich household wealth, older maternal age, adequate ANC, female sex, older child age, and larger birth size are protective, whereas twin births, high family members, higher birth order, fever, and limited access to health services increase risk [13, 14, 16, 21]. Stunting, underweight, diarrhea, febrile illness, and lack of media exposure have also been consistently associated with higher odds of anemia [13, 15, 17].

For decades, anemia among children aged 6–59 months has been defined as a hemoglobin concentration below 11.0 g/dL, based on earlier WHO recommendations [25]. However, accumulating evidence has indicated that these long‐standing thresholds may misclassify anemia status and, in some populations, overestimate anemia prevalence [26]. Consequently, the WHO released updated guidelines in 2024, providing revised hemoglobin cutoff values and age‐specific normative statements for defining anemia across different population groups, including young children [27, 28].

These updated recommendations were developed through systematic reviews of the available evidence and the application of rigorous, evidence‐based methods, with the aim of improving diagnostic accuracy and enhancing the global comparability of anemia estimates [28].

Although numerous studies have examined the prevalence and determinants of anemia among young children in sub‐Saharan Africa, the vast majority rely on outdated hemoglobin cutoff values derived from earlier WHO guidance. To date, there is a critical lack of region‐wide, multi‐country evidence applying the 2024 WHO anemia thresholds using nationally representative data. Consequently, the true magnitude of anemia and its associated factors among children aged 6–23 months under the revised diagnostic framework remains poorly understood. Applying the updated WHO hemoglobin cutoffs is essential for generating policy‐relevant prevalence estimates and for aligning national and regional monitoring efforts with current global standards.

Therefore, this study aims to assess the prevalence and associated factors of anemia among children aged 6–23 months in sub‐Saharan Africa using the 2024 WHO hemoglobin cutoff, drawing on nationally representative DHS data. The findings are expected to provide updated and robust evidence to inform child nutrition policies and targeted interventions across the region.

## 2 Methods

### 2.1 Study Design, Setting, and Data Source

A cross‐sectional study was conducted using secondary data from the most recent DHS in nine sub‐Saharan African countries with available and comparable data: Burkina Faso, Democratic Republic of Congo, Côte d'Ivoire, Ghana, Lesotho, Madagascar, Mali, Mozambique, and Tanzania. The DHS data were accessed through the DHS Program database (https://www.dhsprogram.com) after obtaining the required permission. DHS surveys are nationally representative household surveys that collect standardized information on population health, nutrition, and demographic characteristics using comparable methodologies across countries. The surveys employ a stratified two‐stage cluster sampling design and are conducted periodically in LMICs. Data were drawn from surveys conducted between 2021 and 2024.

### 2.2 Analytical Sample Selection and Sample Size

The analytical sample was derived from pooled DHS datasets. Initially, a total of 448,000 children under 5 years of age were identified across the included surveys prior to applying eligibility criteria. Children who were deceased at the time of the survey were excluded (*n* = 39,094), restricting the analysis to living children (*n* = 408,906). Children outside the age range of 6–23 months were then excluded (*n* = 378,411), leaving 30,495 children aged 6–23 months. Children with missing hemoglobin measurements were also excluded (*n* = 15,501). The final unweighted analytic sample comprised 14,994 children aged 6–23 months, with a weighted sample of 14,480 children.

### 2.3 Sampling Procedure and Weighting

DHS surveys use a two‐stage sampling procedure. In the first stage, enumeration areas (clusters) are selected with probability proportional to size. In the second stage, households are systematically selected within each cluster. To account for unequal probabilities of selection and non‐response, sampling weights provided by DHS were applied in all analyzes. The complex survey design, including clustering and stratification, was accounted for to ensure valid population‐level estimates.

### 2.4 Variables

#### 2.4.1 Outcome Variable

The outcome variable was anemia status among children aged 6–23 months, measured using hemoglobin concentration adjusted for altitude, as defined by the WHO. Anemia was coded as an ordinal variable with four ordered categories: (i) severe anemia, (ii) moderate anemia, (iii) mild anemia and (iv) no anemia, reflecting increasing levels of hemoglobin concentration.

### 2.5 Explanatory Variables

Explanatory variables were selected based on prior literature and data availability and were categorized into individual‐level and community‐level factors. Individual‐level variables included child, maternal, and household characteristics. Community‐level variables, representing contextual factors, were derived either by aggregating individual‐level characteristics within clusters (community literacy, community media exposure) or obtained directly from DHS data without aggregation (place of residence, distance to health facilities). Child‐level variables included age (6–11 months, 12–23 months), sex (male, female), birth type (single, multiple), birth weight (low < 2500 g, normal 2500–3999 g, and high ≥ 4000 g), perceived birth size (small, average, large), EBF (no, yes), deworming status (no, yes), vitamin A supplementation (no, yes), minimum meal frequency (MMF) (no, yes), minimum dietary diversity (< 4, ≥ 4 food groups), nutritional status (stunted, underweight, wasted; no/yes), and recent illnesses, including diarrhea and fever (no/yes). Maternal and household‐level variables included maternal age (15–24, 25–34, and 35–49 years), maternal education (no education, primary, secondary or higher), maternal literacy (illiterate, literate), employment status (unemployed, employed), marital status (unmarried, married), sex of household head (male, female), media exposure (not exposed, exposed), wealth index (poor, middle, rich), household size (1–4, ≥ 5 members), number of under‐five children (none/one, two, three or more), health insurance coverage (no, yes), ANC visits (inadequate < 4, adequate ≥ 4), postnatal care (no, yes), and place of delivery (home, health facility). Community‐level variables included community literacy and media exposure (low, high), distance to health facilities (big problem, not a big problem), and place of residence (urban, rural).

### 2.6 Operational Definition and Measurement of Child Anemia

Child anemia was assessed using hemoglobin measurements collected during the DHS biomarker survey. Hemoglobin concentrations were adjusted for altitude following World Health Organization (WHO) recommendations. In this study, the DHS variable *hw56*, which contains hemoglobin values already adjusted for altitude, was used. Based on the updated WHO age‐specific cutoffs for children aged 6–23 months, anemia status was classified into four categories: severe (Hb < 7.0 g/dL), moderate (Hb 7.0–9.4 g/dL), mild (Hb 9.5–10.4 g/dL), and no anemia (Hb ≥ 10.5 g/dL) [29, 30]. For the analysis, anemia status was treated as an ordinal outcome variable and coded as follows: (i) severe anemia, (ii) moderate anemia, (iii) mild anemia and (iv) no anemia [4], with higher values indicating less severe anemia. This coding scheme was used in the proportional odds (ordinal logistic regression) model, where the estimated adjusted odds ratios (AORs) represent the likelihood of being in a higher (less severe) anemia category versus all lower (more severe) categories.

### 2.7 Operationalization and Classification of Some Independent Variables

#### 2.7.1 Maternal Literacy

Maternal literacy was assessed using the DHS literacy variable (v155). Women who were able to read part or all of a sentence were classified as literate, while those who could not read at all were classified as illiterate.

#### 2.7.2 Maternal Marital Status

Marital status was determined using the DHS variable v502 and classified as married/living with a partner or not married (never married, widowed, divorced, or separated).

#### 2.7.3 Maternal Employment Status

Employment status was derived from the DHS variable v714 and categorized as employed (engaged in any form of work) or not employed (not engaged in any work).

#### 2.7.4 Birth Weight

Birth weight was obtained from DHS variable m19. Children were classified as low birth weight (< 2500 g), normal birth weight (2500–3999 g), or high birth weight (≥ 4000 g) according to WHO guidelines.

#### 2.7.5 EBF

EBF was defined as infants aged 0–5 months that were currently breastfed and had not received any other liquids or foods, except for medicines and supplements. Infants who were not currently breastfed or who had received any other foods or liquids were classified as not exclusively breastfed.

#### 2.7.6 MMF

MMF was assessed using DHS data (m4, m38, m39, b19) based on breastfeeding status, age, and number of meals in the previous 24 h. Breastfed children aged 6–8 months were considered to meet MMF if they received ≥ 2 meals/day, those aged 9–23 months if ≥ 3 meals/day, and non‐breastfed children aged 6–23 months if ≥ 4 total feeds/day. Children not meeting these thresholds were classified as not meeting MMF.

##### 2.7.6.1 Minimum Dietary Diversity (MDD)

MDD was assessed among children aged 6–23 months in accordance with WHO Infant and Young Child Feeding guidelines. Dietary diversity was determined based on maternal report of the child's consumption of seven standard food groups in the 24 h preceding the survey: grains, roots and tubers (v414e); legumes and nuts (v414f); dairy products (v414g); flesh foods (v414h); eggs (v414i); vitamin A–rich fruits and vegetables (v414j); and other fruits and vegetables (v414k). Children who consumed foods from at least four of the seven food groups were classified as having met minimum dietary diversity.

##### 2.7.6.2 Child Nutritional Status

Child nutritional status was assessed using anthropometric indicators based on the WHO growth standards, as reported in the DHS datasets. Stunting was defined using height‐for‐age z‐scores (HAZ, DHS variable hw70) as HAZ < –2 standard deviations (SD) from the WHO median. Underweight was defined using weight‐for‐age z‐scores (WAZ, DHS variable hw71) as WAZ < –2SD. Wasting was defined using weight‐for‐height z‐scores (WHZ, DHS variable hw72) as WHZ < –2SD.

### 2.8 Statistical Analysis

All statistical analyzes were conducted using Stata version 17. Sample weight was applied in all analyzes to account for unequal probabilities of selection, and clustering and stratification were incorporated to reflect the DHS survey design. Descriptive statistics were used to summarize the characteristics of the study population. Given the ordinal nature of anemia severity and the hierarchical structure of the DHS data, a multilevel proportional odds (ordinal logistic) regression model with random intercepts at the cluster (community) level was employed [31, 32]. Anemia severity was classified into ordered categories based on the updated WHO hemoglobin cut‐offs (severe, moderate, mild, and no anemia), justifying the use of an ordinal modeling approach. Variables with a *p*‐value less than 0.2 in the bivariate analysis were included in the multivariable analysis. A two‐level model was fitted, with children nested within communities (primary sampling units), to account for intra‐cluster correlation and unobserved cluster‐level heterogeneity. Four models were estimated: a null model with no predictors, a model including individual‐level variables, a model including community‐level variables, and a combined model incorporating both individual‐ and community‐level factors. Model fit was evaluated using the log‐likelihood, deviance, Akaike Information Criterion (AIC), and Bayesian Information Criterion (BIC).

The general form of the fitted model was:

$$\log\left(\text{Pr}\left(Y_{\text{ij}}\leq k\right)/\text{Pr}\left(Y_{\text{ij}}>k\right)\right)=\alpha_{k}+{\beta X}_{\text{ij}}+u_{j},$$

where *Y_ij_* denotes anemia severity for child i in community *j*; *α_k_* are category‐specific thresholds; *β* are fixed‐effect coefficients for individual‐ and community‐level covariates; and *u_j_* is a random intercept representing between‐community variability, assumed to follow a normal distribution with mean zero and variance σ^2^_u_ [33, 34]. The analysis was implemented using the meologit command in Stata, with sample weight specified as analytic weight and clustering defined at the community level (v021) [35]. Results were reported as AORs with corresponding 95% confidence intervals (CIs). The estimated AORs represent the odds of being in a higher (less severe) anemia category versus all lower (more severe) categories under the proportional odds assumption [31, 32]. An AOR greater than 1 indicates a higher likelihood of being in less severe anemia categories, whereas an AOR less than 1 indicates a higher likelihood of more severe anemia. Statistical significance was assessed using a two‐sided *p*‐value of < 0.05.

### 2.9 Model Assumptions

Anemia severity was analyzed as an ordinal outcome with four ordered categories. The proportional odds (parallel lines) assumption was evaluated prior to multilevel modeling using a single‐level generalized ordered logistic regression with the gologit2 command and the autofit option [36, 37], as formal tests are not available after multilevel estimation. Given the hierarchical structure of the DHS data, a multilevel proportional odds regression model with random intercepts at the cluster level was fitted for inference. Model adequacy was additionally examined by assessing whether adjusted predicted probabilities followed the expected ordinal pattern across anemia categories.

### 2.10 Assessment of Cluster‐Level Variation

To quantify between‐cluster variation in childhood anemia, the intraclass correlation coefficient (ICC), median odds ratio (MOR), and proportional change in variance (PCV) were computed, which are standard measures in multilevel analysis for assessing clustering effects. The ICC was used to quantify the proportion of total variation in childhood anemia attributable to differences between clusters across the fitted models. The ICC was estimated for each model directly from the multilevel proportional odds (ordinal logistic regression) model using the Stata post‐estimation command estat icc, which derives the ICC from the estimated cluster‐level random‐effects variance under the ordinal logistic distributional assumption. A higher ICC value indicates greater similarity of outcomes within clusters and stronger contextual (community‐level) effects. The MOR was calculated to express the magnitude of cluster‐level heterogeneity on the odds ratio scale. The MOR represents the median increase in the odds of childhood anemia when moving from a lower‐risk cluster to a higher‐risk cluster for two children with identical individual‐level characteristics. An MOR value of 1 indicates the absence of between‐cluster variation. The MOR was derived from the estimated cluster‐level variance using the following formula: MOR = exp[0.6745 × √(2 × σ^2^ᵤ)], where σ^2^ᵤ denotes the cluster‐level variance.

The PCV measures the extent to which the inclusion of explanatory variables reduces the cluster‐level variance compared with the null model. It indicates how much of the between‐cluster variation in childhood anemia is explained by the variables included in the model. Higher PCV values reflect greater explanatory power. Formula: PCV = ((σ^2^_u0_ − σ^2^_u1_)/σ^2^_u0_) × 100, where: σ^2^_u0_ is the cluster‐level variance in the null model, and σ^2^_u1_ is the cluster‐level variance in the adjusted model.

### 2.11 Handling of Missing Data

Missing data were assessed for all study variables prior to analysis. Occasional missingness occurred for ANC and postnatal care, while birth weight, birth size, vitamin A supplementation, deworming, place of delivery, maternal literacy, fever, and diarrhea had minimal missing values. Given the low overall missingness (approximately 3% for ANC and postnatal care and less than 1% for other variables) and the absence of systematic patterns, complete case analysis was used. Multiple imputation was not performed, as it was deemed unnecessary under these conditions.

### 2.12 Ethical Considerations

This study was based on secondary analysis of publicly available DHS data. Ethical approval for the DHS surveys is obtained by the implementing agencies in each country, and informed consent is obtained from all participants. Permission to access and use DHS data was obtained from the DHS Program following submission of a research proposal. No additional ethical approval was required. The DHS datasets are publicly accessible at https://dhsprogram.com.

## 3 Results

### 3.1 Sociodemographic and Health Characteristics of Study Population

The study included 14,994 children aged 6–23 months, corresponding to a weighted sample of 14,480 children after applying survey weights to ensure representativeness. Two‐thirds of the children were aged 12–23 months (66.6%), and slightly more than half were male (51%). Most mothers were aged 25–34 years (42.8%), and the majorities were married (86.9%). Nearly 60% of mothers were employed, and over two‐thirds of households resided in rural areas (71.3%). About 36.1% of mothers had no formal education, while nearly two‐thirds were illiterate (65.5%). Most households were male‐headed (80.3%), and 60.8% of mothers were exposed to media. Most households had a large family size, with 93.5% having five or more members, while 39.4% of households had two children under the age of five. Nearly half of households (44.8%) were in the poor wealth index, and more than one‐third of respondents (35.4%) reported distance to a health facility as a big problem. Only 11.9% of households had health insurance. Nearly one‐fifth (22.8%) of women delivered at home, and most births were single (96.9%). About one‐quarter of women utilized postnatal care services (24.9%), and 40% of mothers had inadequate ANC attendance (< 4 ANC visits). EBF was reported for only 9% of children. About two in five children (38.3%) had received deworming, while just over half (54.2%) had been given vitamin A supplementation. Low birth weight was observed in 6.9% of children, and 11.7% were of small birth size. About 93.2% of children did not meet the MMF, and 92.5% did not achieve minimum dietary diversity. The prevalence of stunting, underweight, and wasting was 29.8%, 18.5%, and 9.1%, respectively. Additionally, 21.9% of children experienced diarrhea, and 20.8% had fever during the reference period. At the community level, nearly half of the mothers lived in communities with low literacy (49.1%), while 53.1% resided in communities with high media exposure (Table 1).

**Table 1:** Sociodemographic and health characteristics of the study population.

Variables	Category	Weighted frequency	Percentages
Child age	6–11 months	4831	33.4%
	12–23 months	9649	66.6%
Child sex	Males	7380	51%
	Females	7100	49%
Maternal age (in years)	15–24	5248	36.3%
	25–34	6200	42.8%
	35–49	3032	20.9%
Marital status	Unmarried	1897	13.1%
	Married	12,583	86.9%
Maternal employment status	Unemployed	5807	40.1%
	Employed	8673	59.9%
Resident	Urban	4159	28.7%
	Rural	10,321	71.3%
Maternal education	No education	5229	36.1%
	Primary	4234	29.2%
	Secondary and higher	5017	34.7%
Maternal literacy	Illiterates	9461	65.5%
	Literates	4975	34.5%
Sex of household head	Males	11,635	80.3%
	Females	2845	19.7
Media exposure	Not exposed	5671	39.2%
	Exposed	8809	60.8%
Wealth index	Poor	6487	44.8%
	Middle	2989	20.6%
	Rich	5004	34.6%
House hold size	1–4	971	6.5%
	5 and above	14,023	93.5%
Number of under five children	None or one	4918	34%
	Two	5709	39.4%
	Three and above	3853	26.6%
Distance to health facility	Big problem	5131	35.4%
	Not big problem	9349	64.6%
Place of delivery	Home	3271	22.8%
	Health facility	11,079	77.2%
Birth type	Single	14,032	96.9%
	Multiple	448	3.1%
Health insurance	No	12,764	88.1%
	Yes	1716	11.9%
Exclusive breastfeeding	No	13,169	91%
	Yes	1311	9%
Antenatal care visits	Inadequate (< 4 visits)	5547	40%
	Adequate (≥ 4 visits)	8303	60%
Postnatal care	No	10,430	75.1%
	Yes	3460	24.9%
Children's deworming status	No	8866	61.7%
	Yes	5500	38.3%
Children's Vitamin A supplements	No	6587	45.8%
	Yes	7795	54.2%
Child birth weight	Low (< 2500 g)	1001	6.9%
	Normal (2500–3999 g)	7703	53.3%
	High (≥ 4000 g)	5763	39.8%
Child birth size	Small	1640	11.7%
	Average	7175	51.1%
	Large	5226	37.2%
Children's meet Minimum meal frequency (MMF)	No	13,501	93.2%
	Yes	979	6.8%
Children's minimum dietary diversity	< 4 food groups	13,400	92.5%
	≥ 4 food groups	1080	7.5%
Stunted	No	10,171	70.2%
	Yes	4309	29.8%
Underweight	No	11,805	81.5%
	Yes	2675	18.5%
Wasting	No	13,167	90.9%
	Yes	1313	9.1%
Diarrhea	No	11,307	78.1%
	Yes	3165	21.9%
Fever	No	11,458	79.2%
	Yes	3014	20.8%
Community literacy	Low	7110	49.1%
	High	7370	50.9%
Community media exposure	Low	6790	46.9%
	High	7690	53.1%

### 3.2 Prevalence of Anemia by Severity Among Children Aged 6–23 Months in Nine Sub‐Saharan African Countries

**Figure 1:**
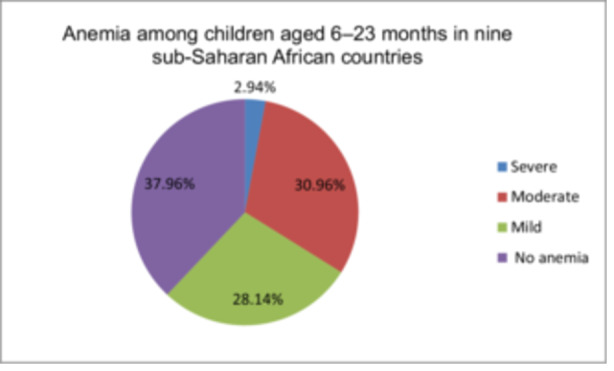
Prevalence of anemia by severity among children aged 6–23 months in nine Sub‐Saharan African countries.

Among children aged 6–23 months in the included sub‐Saharan African countries, 62.04% (95% CI: 61.3–62.8) were anemic, including 2.94% (95% CI: 2.68–3.23) severe, 31.96% (95% CI: 30.21–31.72) moderate, and 28.14% (95% CI: 27.41–28.88) mild anemia, while 37.96% (95% CI: 37.17–38.75) were not anemic (Figure 1).

Moderate anemia was the most common form of anemia in most countries, particularly in Mali (41.8%), Burkina Faso (41.4%), and Mozambique (34.5%). Mild anemia was also common, affecting roughly one‐quarter to one‐third of children across all countries, with the highest prevalence observed in Mozambique (31.2%) and Mali (29.5%). The prevalence of severe anemia was comparatively low across countries but remained notable in Mozambique (7.2%) and Côte d'Ivoire (5.4%). In contrast, Ghana and Madagascar reported the lowest levels of severe anemia (1.2% and 0.7%, respectively) (Table 2).

**Table 2:** Prevalence of anemia severity among children aged 6–23 months in nine sub‐Saharan African countries.

Country	Weighted frequency	Severe (%)	Moderate (%)	Mild (%)	No anemia (%)
Burkina Faso	1686	3.1	41.4	27.9	27.6
DRC	2953	3.2	28.0	29.3	39.5
Côte d'Ivoire	1338	5.4	31.8	26.5	36.3
Ghana	1355	1.2	18.9	24.3	55.6
Lesotho	322	0.5	26.3	27.2	46.0
Madagascar	1828	0.7	18.9	29.1	51.3
Mali	2182	2.2	41.8	29.5	26.5
Mozambique	1218	7.2	34.5	31.2	27.1
Tanzania	1598	2.6	32.1	25.8	39.5

Abbreviation: DRC, Democratic Republic of Congo.

### 3.3 Results of Missing Data Extent and Pattern

The missing data across the variables is generally very low. ANC and postnatal care each have approximately 3% missing values, often occurring together, suggesting a slight joint missing pattern. Other variables, including birth weight, birth size, vitamin A supplementation, deworming, and place of delivery, have less than 1% missing values with scattered, occasional patterns. Maternal literacy, fever, and diarrhea show rare missingness, also below 1%. Overall, the proportion of missing data is minimal, and although ANC and postnatal care show some joint missingness, it is still relatively small. Given these low levels of missingness, a complete case analysis is appropriate and unlikely to introduce significant bias or reduce precision, while multiple imputation is not necessary.

### 3.4 Multicollinearity Assessment Results

The assessment of multicollinearity using the variance inflation factor (VIF) indicated that there was no significant multicollinearity among the independent variables included in the model. VIF values ranged from 1.01 to 1.73, with a mean VIF of 1.25, all well below the commonly used thresholds of 5 or 10. This suggests that none of the variables were highly correlated with each other, and therefore, including all variables in the regression model is appropriate.

### 3.5 Model Assumptions and Justification

The proportional odds (parallel lines) assumption was evaluated prior to multilevel modeling using a single‐level generalized ordered logistic regression fitted with the gologit2 command and the autofit option, as formal tests are not available after multilevel estimation. The global Wald test was not statistically significant (χ^2^ = 63.26, df = 64, *p* = 0.503), indicating no overall violation of the proportional odds assumption. Although most covariates satisfied the assumption, a small number of variables including maternal age, media exposure, deworming, child age, fever, distance to the health facility, household size, and underweight status showed evidence of non‐proportional effects, suggesting variation in their associations across anemia severity thresholds. Model adequacy was further supported by the ordered pattern of adjusted predicted probabilities across anemia categories, with estimates of 2.7% (95% CI: 2.4%–2.9%) for severe anemia, 31.4% (95% CI: 30.6%–32.1%) and 28.9% (95% CI: 28.2%–29.7%) for the intermediate categories, and 37.0% (95% CI: 36.2%–37.9%) for the non‐anemic category. The narrow CIs and large sample size indicate stable estimation. Given the absence of a global violation of the proportional odds assumption and the limited non‐proportionality observed among a small subset of predictors, a multilevel proportional odds regression model with random intercepts at the community level was considered appropriate and used for inference, accounting for clustering of children within communities.

### 3.6 Model Fitness

Model fitness statistics indicated a substantial improvement after the inclusion of individual‐level variables. The null model showed the poorest fit (log‐likelihood = −17,153.3; deviance = 34,306.6; AIC = 34,314.7; BIC = 34,345.2). The individual‐level model markedly improved model fit (log‐likelihood = −14,979.8; deviance = 29,959.6; AIC = 30,045.6; BIC = 30,368.8), whereas the community‐level model showed little improvement over the null model (log‐likelihood = −17,094.1; deviance = 34,188.1; AIC = 34,204.1; BIC = 34,265.1). The combined individual and community‐level model achieved the lowest AIC (30,040.3), with a log‐likelihood (−14,981.2) and a slightly lower BIC (30,333.4) compared with the individual‐level model. Overall, these results indicate that childhood anemia is primarily explained by individual‐level factors; however, the inclusion of community‐level variables provides a modest improvement, suggesting that the combined (individual‐ and community‐level) model offers some added value for inference (Table S1).

### 3.7 Measure of Variation

The random effects analysis indicated that most of the variation in the outcome occurred at the individual level, with only a small proportion attributable to clustering at the community level. Specifically, the ICC ranged from 4.14% to 4.44% across models, suggesting modest similarity among individuals within the same cluster. The MOR ranged from 1.43 to 1.45, indicating that moving from a lower‐ to higher‐risk cluster increased the median odds of the outcome by approximately 1.4 times due to community‐level differences. The PCV showed that individual‐level covariates explained 6.6% of the variance, while community‐level covariates accounted for only 2.4%, and the combined model explained 7.0%. These findings indicate that individual‐level factors were the main contributors to variation, whereas community‐level factors had minimal influence within the DHS hierarchical structure (Table S1).

### 3.8 Factors Associated With Anemia Among Children Aged 6–23 Months

The multilevel proportional odds model indicated that anemia severity among children aged 6–23 months was primarily associated with individual‐level factors, with limited variation attributable to community‐level clustering. Under the proportional odds assumption, AORs represent the likelihood of being in higher (less severe) anemia categories versus lower (more severe) categories.

Female children had higher odds of being in less severe anemia categories compared with males (AOR = 1.40, 95% CI: 1.27–1.45). Children aged 12–23 months had lower odds of being in less severe anemia categories compared with those aged 6–11 months (AOR = 0.90, 95% CI: 0.84–0.97), indicating greater likelihood of more severe anemia. Multiple births (AOR = 0.65, 95% CI: 0.49–0.85), recent fever (AOR = 0.75, 95% CI: 0.67–0.81), and underweight status (AOR = 0.87, 95% CI: 0.78–0.97) were also associated with higher likelihood of more severe anemia. Conversely, normal (AOR = 1.21, 95% CI: 1.05–1.40) and high birth weight (AOR = 1.23, 95% CI: 1.06–1.44) were associated with higher odds of being in less severe anemia categories. Maternal age, education, literacy, and employment were similarly associated with higher odds of less severe anemia. Children from wealthier households and those with maternal health insurance coverage also had higher odds of being in less severe anemia categories. Additionally, deworming (AOR = 1.14, 95% CI: 1.06–1.23), adequate ANC (AOR = 1.09, 95% CI: 1.01–1.17), and minimum dietary diversity (AOR = 1.25, 95% CI: 1.10–1.41) were associated with less severe anemia. Several variables, including stunting, wasting, diarrhea, postnatal care, media exposure, and vitamin A supplementation, were not significantly associated with anemia severity. Detailed fixed‐effect estimates for both significant and non‐significant variables across the four models are provided in Table S1, while the fully adjusted model (including both individual‐ and community‐level factors), with only significant variables, is presented in Table 3.

**Table 3:** Fully adjusted multilevel model (individual‐ and community‐level factors) associated with anemia among children aged 6–23 months.

Variables	Category	Individual and community level
		AOR (95% CI)
Child age	6–11 months	1
	12–23 months	**0.9 (0.84–0.97)**
Child sex	Males	1
	Females	**1.4 (1.27–1.45)**
Maternal age (in years)	15–24	1
	25–34	**1.17 (1.09–1.27)**
	35–49	**1.36 (1.24–1.5)**
Maternal employment status	Unemployed	1
	Employed	**1.16 (1.08–1.24)**
Maternal education	No education	1
	Primary	**1.32 (1.21–1.45)**
	Secondary and higher	**1.37 (1.23–1.52)**
Maternal literacy	Illiterates	1
	Literates	**1.17 (1.07–1.28)**
Wealth index	Poor	1
	Middle	1.042 (0.95–1.14)
	Rich	**1.11 (1.02–1.22)**
Number of under five children	None or one	1
	Two	0.94 (0.87–1.01)
	Three and above	**0.74 (0.67–0.81)**
Birth type	single	1
	Multiple	**0.65 (0.49–0.85)**
Health insurance	No	1
	Yes	**1.74 (1.55–1.96)**
Antenatal care (ANC) visits	Inadequate(< 4 ANC)	1
	Adequate (≥ 4 ANC)	**1.09 (1.013–1.17)**
Children's deworming status	No	1
	Yes	**1.14 (1.06–1.23)**
Child birth weight	Low (< 2500 g)	1
	Normal (2500–3999 g)	**1.21 (1.05–1.4)**
	High (≥ 4000 g)	**1.23 (1.06–1.44)**
Minimum dietary diversity	< 4 food groups	1
	≥ 4 food groups	**1.25 (1.1–1.41)**
Underweight	No	1
	Yes	**0.87 (0.78–0.97)**
Fever	No	1
	Yes	**0.75 (0.67–0.81)**

*Note:* Anemia was coded as an ordinal variable (1 → severe, 2 → moderate, 3 → mild, 4 → no anemia), and a proportional odds (ordinal logistic) model was applied. Adjusted odds ratios (AORs) represent the odds of being in a higher (less severe) anemia category versus all lower (more severe) categories under the proportional odds assumption. An AOR < 1 indicates higher odds of more severe anemia, whereas an AOR > 1 indicates higher odds of less severe anemia. Statistically significant associations (*p* < 0.05% and 95% CI that do not include 1) are presented in bold. Only statistically significant variables are presented in the final model (Table 3), while all variables, including non‐significant ones, are reported in Table S1.

## 4 Discussion

Earlier WHO hemoglobin cutoffs for young children were based on limited historical data and did not fully account for physiological variations in hemoglobin levels during early childhood. As a result, these thresholds may have classified some children with normal hemoglobin levels as anemic, leading to overestimation of anemia prevalence. The updated 2024 WHO thresholds are based on improved evidence and more accurately reflect age‐specific physiological norms, thereby reducing misclassification and providing a more precise estimate of the true burden. This study provides updated evidence on the burden and severity of anemia among children aged 6–23 months in the included sub‐Saharan African countries using the revised 2024 WHO hemoglobin cutoffs. Despite these updated thresholds, anemia remains a major public health problem, affecting 62% of children in this age group. Although this estimate is lower than previous reports based on earlier WHO cutoffs [16, 20, 24], it remains substantial and supports the evidence that earlier WHO thresholds may have overestimated anemia prevalence [13, 14, 15, 17, 20, 21, 22] due to misclassification of physiologically normal hemoglobin levels as anemia. Importantly, the persistence of a high burden under the revised criteria indicates that childhood anemia reflects genuine underlying challenges, including poor nutrition, infectious diseases, and socioeconomic disadvantage [17], rather than solely a measurement issue.

Moderate anemia was the most common severity category across countries, suggesting widespread chronic nutritional deficiencies and sustained exposure to contributing factors such as poor dietary quality and recurrent infections [4, 5, 16, 20, 23]. Although severe anemia was less common, its presence in certain countries remains concerning due to its strong association with increased morbidity, mortality, and impaired child development [4, 5, 6]. Even relatively small proportions of severe anemia represent a critical public health concern, particularly in resource‐limited settings [1, 2, 3]. The observed variation in anemia prevalence and severity across countries highlights important contextual differences in socioeconomic conditions, food security, disease burden, and health system performance [14, 17, 18, 19]. Higher anemia levels are likely linked to structural challenges such as poverty, limited access to diverse diets, and inadequate maternal and child health services [13, 22, 23]. In contrast, relatively lower severity in some settings may reflect improvements in maternal education, infant feeding practices, and primary healthcare access [16, 23].

The findings of this study indicate that anemia severity among children aged 6–23 months is influenced by a combination of child, maternal, and household‐level factors. Under the proportional odds assumption, AORs represent the likelihood of being in a higher (less severe) anemia category versus all lower (more severe) categories combined. Accordingly, an adjusted odds ratio greater than 1 indicates a higher likelihood of being in less severe anemia categories, whereas an adjusted odds ratio less than 1 indicates a higher likelihood of being in more severe anemia categories.

Female children were more likely to be in less severe anemia categories compared with males, suggesting a higher burden of severe anemia among male children. This finding is consistent with previous studies reporting greater vulnerability among male children [13, 16, 19]. The observed sex difference may be explained by higher growth velocity and iron requirements among male infants, which increase susceptibility to iron deficiency when dietary intake is inadequate [7, 8, 23]. Older children (12–23 months) were more likely to experience more severe anemia, which may reflect increased iron requirements during rapid growth alongside suboptimal dietary intake [7, 8]. Multiple births and larger numbers of young children in the household were also associated with greater anemia severity, possibly reflecting resource constraints and caregiving challenges. This finding is consistent with previous studies reporting that multiple births and larger household sizes are associated with increased risk of childhood anemia [13, 14, 16, 22]. Recent fever was associated with more severe anemia, suggesting increased severity of anemia among children with recent illness. This finding is consistent with previous studies [13, 14, 15, 16] and supports existing evidence linking infectious diseases to anemia through inflammation‐induced iron sequestration and impaired erythropoiesis [4, 5, 20]. These results highlight the close interrelationship between infectious diseases and anemia, likely mediated by inflammatory processes that lead to iron sequestration, disrupted iron metabolism, and impaired red blood cell production. Similarly, underweight status was associated with increased anemia severity, reflecting the close relationship between undernutrition and hematological health [38]. This finding supports previous evidence that undernutrition and anemia frequently coexist [13, 15], as both conditions share common pathways, including inadequate dietary intake, recurrent infections, impaired nutrient absorption, and weakened immunity [39].

In contrast, higher maternal education, literacy, and employment, as well as greater household wealth and health insurance coverage, were associated with less severe anemia. These associations likely reflect better access to healthcare, improved child feeding practices, and stronger caregiving capacity. This finding is consistent with DHS‐based studies showing that maternal educational and socioeconomic advantages are linked to lower childhood anemia, particularly in disadvantaged settings [13, 14, 15, 16, 17, 19, 21, 22]. Educated and economically empowered mothers are more likely to secure nutritious foods, obtain timely treatment, and utilize preventive health services, thereby reducing anemia severity among children. Adequate ANC, deworming, and dietary diversity were also associated with less severe anemia, highlighting the importance of maternal and child health services and nutrition‐related factors [6, 13, 16, 23].

### 4.1 Strengths and Limitations of Study

This study has several strengths. It used large, nationally representative samples from nine included sub‐Saharan African countries, allowing for robust population‐level estimates. The data were drawn from standardized DHS surveys, ensuring high quality, comparability, and reliability across countries. Applying the updated 2024 WHO hemoglobin cutoffs provided a more accurate assessment of anemia prevalence and severity among children aged 6–23 months. The multilevel modeling approach accounted for the hierarchical structure of the data and quantified both individual‐ and community‐level effects, while adjusting for the complex survey design, including sampling weights, clustering, and stratification. Additionally, the study included a comprehensive set of child, maternal, household, and community‐level variables, identifying actionable programmatic targets to reduce anemia.

However, some limitations should be considered. The cross‐sectional design precludes causal inference. Although missing data were minimal, approximately 3% of observations were missing for antenatal and postnatal care variables, which may introduce slight bias if the missingness is not completely random. The use of secondary DHS data limited the inclusion of important variables such as detailed dietary intake, micronutrient quantities, and genetic conditions like hemoglobinopathies. Additionally, dietary diversity was used as a proxy for nutrient intake; however, this indicator reflects only the variety of food groups consumed and does not capture the quantity, bioavailability, or actual micronutrient content of the diet, which may lead to misclassification of nutritional adequacy. Maternal recall bias may also affect reported child feeding practices and illness history. Furthermore, although multiple countries were included, the findings are based on nine countries and may not be fully generalizable to all sub‐Saharan African settings. Residual confounding may also persist due to unmeasured variables. Finally, measurement variability in hemoglobin assessment and residual confounding from unmeasured factors may have influenced the observed associations.

### 4.2 Public Health Implications

Applying the 2024 WHO hemoglobin cutoffs, this study shows that anemia remains highly prevalent among children aged 6–23 months in the included sub‐Saharan African countries, with substantial levels of moderate and severe cases. While the revised thresholds improve classification accuracy and reduce overestimation, the persistence of a high burden confirms that childhood anemia is a genuine public health problem. These findings support the use of the updated WHO criteria for population monitoring and highlight the need for integrated, multisectoral interventions. Priority strategies include improving maternal nutrition and ANC, promoting women's education and empowerment, expanding dietary diversity, scaling up deworming and infection control, and supporting low‐income households. Coordinated implementation of these measures is essential to reduce anemia severity and improve child health outcomes in resource‐limited settings.

## 5 Conclusion

Anemia among children aged 6–23 months in the included sub‐Saharan African countries remains highly prevalent, with moderate anemia being the most common form and severe anemia persisting in several settings. Several child, maternal, and household‐level factors were found to be associated with anemia severity. Factors such as female sex, adequate dietary diversity, normal or high birth weight, and deworming were associated with lower anemia severity, whereas older age, underweight status, recent fever, multiple births, and a higher number of young children in the household were associated with higher anemia severity. Maternal education, employment, health insurance, ANC, and household wealth were also associated with less severe anemia. These findings highlight the importance of integrated, context‐specific strategies aimed at improving child nutrition, strengthening maternal health services, and addressing socioeconomic disparities.

## Author Contributions

**Temesgen Gebeyehu Wondmeneh:** conceptualization, investigation, funding acquisition, writing – original draft, writing – review and editing, visualization, validation, methodology, software, formal analysis, project administration, resources, supervision, data curation.

## Funding

The author has nothing to report.

## Ethics Statement

Ethical approval procedures were conducted by the respective DHS implementing agencies in each country. This study used publicly available, de‐identified DHS data. Permission to access and use DHS data was obtained from the DHS Program following submission of a research proposal. The analysis adhered to the principles outlined in the Declaration of Helsinki. No individual consent was required for this secondary data analysis.

## Consent

The author has nothing to report.

## Conflicts of Interest

The author declares no conflicts of interest.

## Transparency Statement

The lead/corresponding author (Temesgen Gebeyehu Wondmeneh) affirms that this manuscript is an honest, accurate, and transparent account of the study being reported; that no important aspects of the study have been omitted; and that any discrepancies from the study as originally planned have been clearly explained.

## Supporting information


**Table S1:** All four fixed‐effect models, along with model fit statistics and random effects for all variables.

## Data Availability

For this analysis, the researcher used the DHS dataset obtained from the DHS Program. To request the dataset for research purpose, a research proposal must be submitted to the DHS Program at: https://dhsprogram.com/data/dataset admin/inde x. cf m. The DHS Program typically reviews data requests within 24–48 h and notifies applicants upon approval. Once access is granted, researcher can log in and download the requested data in the preferred format.
