# Supplementary material for: Anemia and Its Associated Factors Among Children Aged 6–23 Months in Nine Sub‐Saharan African Countries: A Multilevel Proportional Odds Model Using the Latest Demographic and Health Survey (DHS) Data
Source: Health Sci Rep. 2026 Jun 19;9(6):e72693. doi: 10.1002/hsr2.72693 (PMC13281421; doi:10.1002/hsr2.72693)
Supplement: Supplementary file 1 — Table S1: All four fixed‐effect models, along with model fit statistics and random effects for all variables. [file HSR2-9-e72693-s001.docx]

Supplementary Table 1 All four fixed-effect models for factors associated with anemia, along with model fit statistics and random effects among children aged 6–23 months in nine sub-Saharan African countries

| Variables | Category | Null model | Individual level | Community level | Individual and community level |
| --- | --- | --- | --- | --- | --- |
|  |  |  | AOR (95% CI) | AOR (95% CI) | AOR (95% CI) |
| Child age | 6-11 months |  | 1 |  | 1 |
|  | 12-23 months |  | **0.9 (0.84-0.97)** |  | **0.9 (0.84-0.97)** |
| Child sex | Males |  | 1 |  | 1 |
|  | Females |  | **1.36 (1.27-1.45)** |  | **1.4 (1.27-1.45)** |
| Maternal age (in years) | 15-24 |  | 1 |  | 1 |
|  | 25-34 |  | **1.18 (1.09-1.27)** |  | **1.17 (1.09-1.27)** |
|  | 35-49 |  | **1.37 (1.24-1.5)** |  | **1.36 (1.24-1.5)** |
| Marital status | Unmarried |  | 1 |  | 1 |
|  | Married |  | 0.99 (0.89-1.1) |  | 0.99 (0.89-1.1) |
| Maternal employment status | Unemployed |  | 1 |  | 1 |
|  | Employed |  | **1.16 (1.08-1.24)** |  | **1.16 (1.08-1.24)** |
| Resident | Urban |  |  | 1 | 1 |
|  | Rural |  |  | **0.72 (0.67-0.79)** | 0.94 (0.86-1.04) |
| Maternal education | No education |  | 1 |  | 1 |
|  | Primary |  | **1.33 (1.21-1.45)** |  | **1.32 (1.21-1.45)** |
|  | Secondary and higher |  | **1.38 (1.24-1.53)** |  | **1.37 (1.23-1.52)** |
| Maternal literacy | Illiterates |  | 1 |  | 1 |
|  | Literates |  | **1.17 (1.07-1.28)** |  | **1.17 (1.07-1.28)** |
| Sex of household head | Males |  | 1 |  | 1 |
|  | Females |  | 1.0 (0.91-1.09) |  | 1.0 (0.91-1.09) |
| Media exposure | Not exposed |  | 1 |  | 1 |
|  | Exposed |  | 1.02 (0.95-1.1) |  | 1.02 (0.94-1.1) |
| Wealth index | Poor |  | 1 |  | 1 |
|  | Middle |  | 1.05 (0.96-1.15) |  | 1.042 (0.95-1.14) |
|  | Rich |  | 1.08 (0.97-1.19) |  | **1.11 (1.02-1.22)** |
| House hold size | 4 and below |  | 1 |  | 1 |
|  | 5 and above |  | 1.11 (0.97-1.28) |  | 1.11 (0.97-1.28) |
| Number of under five children | None or one |  | 1 |  | 1 |
|  | Two |  | 0.94 (0.87-1.01) |  | 0.94 (0.87-1.01) |
|  | Three and above |  | **0.73 (0.67- 0.8)** |  | **0.74 (0.67-0.81)** |
| Distance to health facility | Big problem |  |  | 1 | 1 |
|  | Not big problem |  |  | **1.14 (1.07-1.22)** | 1.03 (0.96-1.11) |
| Place of delivery | Home |  | 1 |  | 1 |
|  | Health facility |  | 0.91 (0.82-1.01) |  | 0.9 (0.82-1.0) |
| Birth type | single |  | 1 |  | 1 |
|  | Multiple |  | **0.65 (0.49-0.85)** |  | **0.65 (0.49-0.85)** |
| Health insurance | No |  | 1 |  | 1 |
|  | Yes |  | **1.76 (1.57-1.97)** |  | **1.74 (1.55-1.96)** |
| Exclusive breastfeeding | No |  | 1 |  | 1 |
|  | Yes |  | 0.98 (0.87-1.1) |  | 0.98 (0.87-1.1) |
| Antenatal care (ANC) visits | Inadequate(< 4 ANC) |  | 1 |  | 1 |
|  | Adequate (≥ 4 ANC) |  | **1.09(1.01-1.17)** |  | **1.09 (1.013-1.17)** |
| Postnatal care | No |  | 1 |  | 1 |
|  | Yes |  | 1.0 (0.93-1.09) |  | 1.0 (0.93-1.09) |
| Children’s deworming status | No |  | 1 |  | 1 |
|  | Yes |  | **1.14 (1.06-1.23)** |  | **1.14 (1.06-1.23)** |
| Children’s Vitamin A supplements | No |  | 1 |  | 1 |
|  | Yes |  | 1.05 (0.98-1.13) |  | 1.05 (0.97-1.13) |
| Child birth weight | Low (<2500g) |  | 1 |  | 1 |
|  | Normal (2500-3999g) |  | **1.21 (1.05-1.4)** |  | **1.21 (1.05-1.4)** |
|  | High (≥ 4000g) |  | **1.23 (1.06-1.43)** |  | **1.23 (1.06-1.44)** |
| Child birth size | Small |  | 1 |  | 1 |
|  | Average |  | 0.99 (0.88-1.11) |  | 0.99 (0.88-1.11) |
|  | Large |  | 0.98 (0.87-1.11) |  | 0.98 (0.87-1.108) |
| Meet minimum meal frequency | No |  | 1 |  | 1 |
|  | Yes |  | 1.18 (1.0-1.39) |  | 1.18 (1.0-1.39) |
| Minimum dietary diversity | < 4 food groups |  | 1 |  | 1 |
|  | ≥ 4 food groups |  | **1.25 (1.1-1.42)** |  | **1.25 (1.1-1.41)** |
| Stunted | No |  | 1 |  | 1 |
|  | Yes |  | 0.95 (0.87-1.03) |  | 0.95 (0.87-1.04) |
| Underweight | No |  | 1 |  | 1 |
|  | Yes |  | **0.87 (0.78-0.97)** |  | **0.87 (0.78-0.97)** |
| Wasting | No |  | 1 |  | 1 |
|  | Yes |  | 0.99 (0.87-1.13) |  | 0.99 (0.87-1.13) |
| Diarrhea | No |  | 1 |  | 1 |
|  | Yes |  | 0.98 (0.9-1.06) |  | 0.98 (0.9-1.06) |
| Fever | No |  | 1 |  | 1 |
|  | Yes |  | **0.75 (0.69-0.81)** |  | **0.75 (0.67-0.81)** |
| Community literacy | Low |  |  | 1 | 1 |
|  | High |  |  | **1.15 (1.05-1.26)** | 1.03 (0.94-1.13) |
| Community media exposure | Low |  |  | 1 | 1 |
|  | High |  |  | 0.97 (0.89-1.06) | 0.98 (0.9-1.08) |
| **Model fitness statistics** | |  |  |  |  |
| Log-likelihood Ratio (LLR) | | -17153.3 | -14979.8 | -17094.1 | -14981.2 |
| Device (-2LLR) | | 34306.6 | 29959.6 | 34188.1 | 29962.3 |
| AIC |  | 34314.7 | 30045.6 | 34204.1 | 30040.3 |
| BIC |  | 34345.2 | 30368.8 | 34265.1 | 30333.4 |
| **Random effect results** | |  |  |  |  |
| ICC | | 4.44% | 4.16% | 4.34% | 4.14% |
| MOR | | 1.45 | 1.43 | 1.45 | 1.43 |
| PCV | | - | 6.6% | 2.4% | 7.0% |

Note: Anemia was coded as an ordinal variable (1 → severe, 2 → moderate, 3 → mild, 4 → no anemia), and a proportional odds (ordinal logistic) model was applied. Adjusted odds ratios (AORs) represent the odds of being in a higher (less severe) anemia category versus all lower (more severe) categories under the proportional odds assumption. An AOR < 1 indicates higher odds of more severe anemia, whereas an AOR > 1 indicates higher odds of less severe anemia. Statistically significant associations (p < 0.05 and 95% CI that do not include 1) are presented in bold. In Supplementary Table 1, both significant and non-significant variables are reported.
